# Intensive phase treatment outcome and associated factors among patients treated for multi drug resistant tuberculosis in Ethiopia: a retrospective cohort study

**DOI:** 10.1186/s12879-019-4411-7

**Published:** 2019-09-18

**Authors:** Teklu Molie, Zelalem Teklemariam, Eveline Klinkenberg, Yadeta Dessie, Andargachew Kumsa, Hussen Mohammed, Adisalem Debebe, Dawit Assefa, Abebe Habte, Ahmed Bedru, Daniel Fiseha, Berhanu Seyoum

**Affiliations:** 1 Dire Dawa Administration Heath Bureau, Dire Dawa, Ethiopia; 2 College of Health and Medical Sciences, Haramaya University, Harar, Ethiopia; 3 KNCV Tuberculosis Foundation, The Hague, The Netherlands; 4 Department of Global Health and Amsterdam Institute for Global Health and Development, Amsterdam University Medical Centers, Amsterdam, The Netherlands; 5 Federal Ministry of Health of Ethiopia, Addis Ababa, Ethiopia; 6 Dire Dawa University, School of Medicine, Dire Dawa, Ethiopia; 7 KNCV Tuberculosis Foundations /USAID/Challenge TB, Addis Ababa, Ethiopia; 8 Armauer Hansen Research Institute (AHRI), Addis Ababa, Ethiopia

**Keywords:** Multi-drug resistance TB, Outcome, Intensive phase, Ethiopia

## Abstract

**Background:**

Multi-drug resistant Tuberculosis (MDR-TB) is a strain of *Mycobacterium tuberculosis* that is resistant to at least Rifampicin and Isoniazid drugs. The treatment success rate for MDR-TB cases is lower than for drug susceptible TB. Globally only 55% of MDR-TB patients were successfully treated. Monitoring the early treatment outcome and better understanding of the specific reasons for early unfavorable and unknown treatment outcome is crucial for preventing the emergence of further drug-resistant tuberculosis. However, this information is scarce in Ethiopia. Therefore, this study aimed to determine the intensive phase treatment outcome and contributing factors among patients treated for MDR-TB in Ethiopia.

**Methods:**

A 6 year retrospective cohort record review was conducted in fourteen TICs all over the country. The records of 751 MDR-TB patients were randomly selected using simple random sampling technique. Data were collected using a pre-tested and structured checklist. Multivariable multinomial logistic regression was undertaken to identify the contributing factors.

**Results:**

At the end of the intensive phase, 17.3% of MDR-TB patients had an unfavorable treatment outcome, while 16.8% had an unknown outcome with the remaining having a favorable outcome. The median duration of the intensive phase was 9.0 months (IQR 8.04–10.54). Having an unfavorable intensive phase treatment outcome was found significantly more common among older age [ARRR = 1.047, 95% CI (1.024, 1.072)] and those with a history of hypokalemia [ARRR = 0.512, 95% CI (0.280, 0.939)]. Having an unknown intensive phase treatment outcome was found to be more common among those treated under the ambulatory care [ARRR = 3.2, 95% CI (1.6, 6.2)], rural dwellers [ARRR = 0.370, 95% CI (0.199, 0.66)], those without a treatment supporter [ARRR = 0.022, 95% CI (0.002, 0.231)], and those with resistance to a limited number of drugs.

**Conclusion:**

We observed a higher rate of unfavorable and unknown treatment outcome in this study. To improve favorable treatment outcome more emphasis should be given to conducting all scheduled laboratory monitoring tests, assignment of treatment supporters for each patient and ensuring complete recording and reporting which could be enhanced by quarterly cohort review. Older aged and rural patients need special attention. Furthermore, the sample referral network should be strengthened.

## Background

Multi-Drug Resistance Tuberculosis (MDR-TB) is a strain of *Mycobacterium tuberculosis* that is resistant to at least rifampicin and isoniazid drugs. MDR-TB occurs either when a person is infected with a resistant strain of *Mycobacterium tuberculosis* (called primary MDR-TB) or when improper or inadequate treatment leads to drug selection of the resistant strain (called acquired MDR-TB) [1]. The possible causes of inadequate treatment include provider and program related factors like inadequate regimens, lack of drug susceptibility testing (DST) and poor access to health care, drug related factors like unavailability of certain drugs and poor storage conditions and patient related factors, like poor adherence and lack of adequate information [2].

Increasing prevalence of Multi-Drug Resistance or Rifampicin Resistance tuberculosis (MDR/RR-TB) represents a global public health emergency [3]. Emergence of Extensively Drug-Resistant TB (XDR-TB) is further increasing the complexity for TB control programs, especially in low income countries [1]. In 2017, an estimated 558,000 people developed MDR/RR-TB worldwide with 8.5% of these being XDR-TB [4]. Ethiopia is among the 30 high MDR- TB burden countries with an estimated 2700 (1700–3700) MDR/RR-TB cases among annually notified TB cases [4]. Till 2018, the country reported seven pre-XDR-TB cases [5]. The 2018 global TB report estimated 2.7% of new TB cases and 14% of previously treated TB cases in Ethiopia were MDR/RR-TB in 2017. In Ethiopia, 2051 MDR/RR-TB cases were enrolled to SLD between 2009 and 2015 [6], lower numbers than estimated.

Treatment outcomes for MDR-TB cases are poorer compared to drug-susceptible TB cases. This is due to medications used in the treatment of MDR-TB which are less effective and associated with a greater number of side effects, also, treatment duration is at least 20 months which can compromise adherence [7–9]. Globally only 55% of patients with MDR/RR-TB in the 2015 cohort were successfully treated, as a result of high mortality and loss to follow-up [4]. Ethiopia is one of the five high MDR-TB burden countries globally that achieved a treatment success rate above 70% [4]. Although above the global average, this is still far below the 90% target set in the end TB strategy.

Early sputum culture conversion to end the intensive phase, is very important to prevent transmission of MDR-TB, reduce hospitalization time, and reduce cost for both patients and the health system. Evidence has shown that delayed sputum conversion is associated with amplifications of drug resistance including XDR-TB [10]. The few published studies that examined sputum conversion at two months among MDR-TB patients showed that the proportion of MDR-TB patients who converted to culture negative after a median time of 2 months of treatment initiation ranged from 77 to 88% [11, 12].

Some studies indicate that associated factors with failing to culture convert and unfavorable treatment outcome are older age, being male, unemployment, prisoner, alcoholism, baseline AFB smear positive, lung cavitation at baseline chest X-ray, resistance to ofloxacin and streptomycin, history of previous TB treatment and poor outcome of previous anti-tuberculosis treatment, smoker, drug user, HIV co-infection, lower body mass index and lower CD4 count [9, 11, 13–16].

There are few studies conducted on intensive phase treatment outcome and contributing factors among MDR-TB patients and none in Ethiopia despite it being one of high MDR-TB burden countries. Gaining insight in the early treatment outcomes could assist the Ethiopian National TB Program to further improve the treatment success rate for MDR-TB patients in the country towards the 90% target of the end TB strategy. At the same time, this study could also serve as a baseline for future broader studies.

Therefore, this study was conducted to determine intensive phase treatment outcome and associated factors among patients treated for MDR-TB in Ethiopia.

## Methods

### Study setting

This study was conducted in a random set of patients from all MDR-TB treatment initiation sites in Ethiopia. The country’s population was estimated at 102 million in 2017, with 84% being rural [17]. In 2018, there were 281 public hospitals, 3622 health centers, and 16,660 health posts in the country. All the hospitals and health centers provided TB diagnosis and treatment services and 65% of health posts provided DOTs service for drug susceptible TB patients [5].

Till 2014, there were 14 MDR-TB TICs (one in Tigrai, three in Amhara, five in Oromia, two in Southern Nations and Nationality Peoples’ Regional States (SNNPR), two in Addis Ababa and one in Dire Dawa City Administrations) found in Ethiopia [6].

**Fig. 1:**
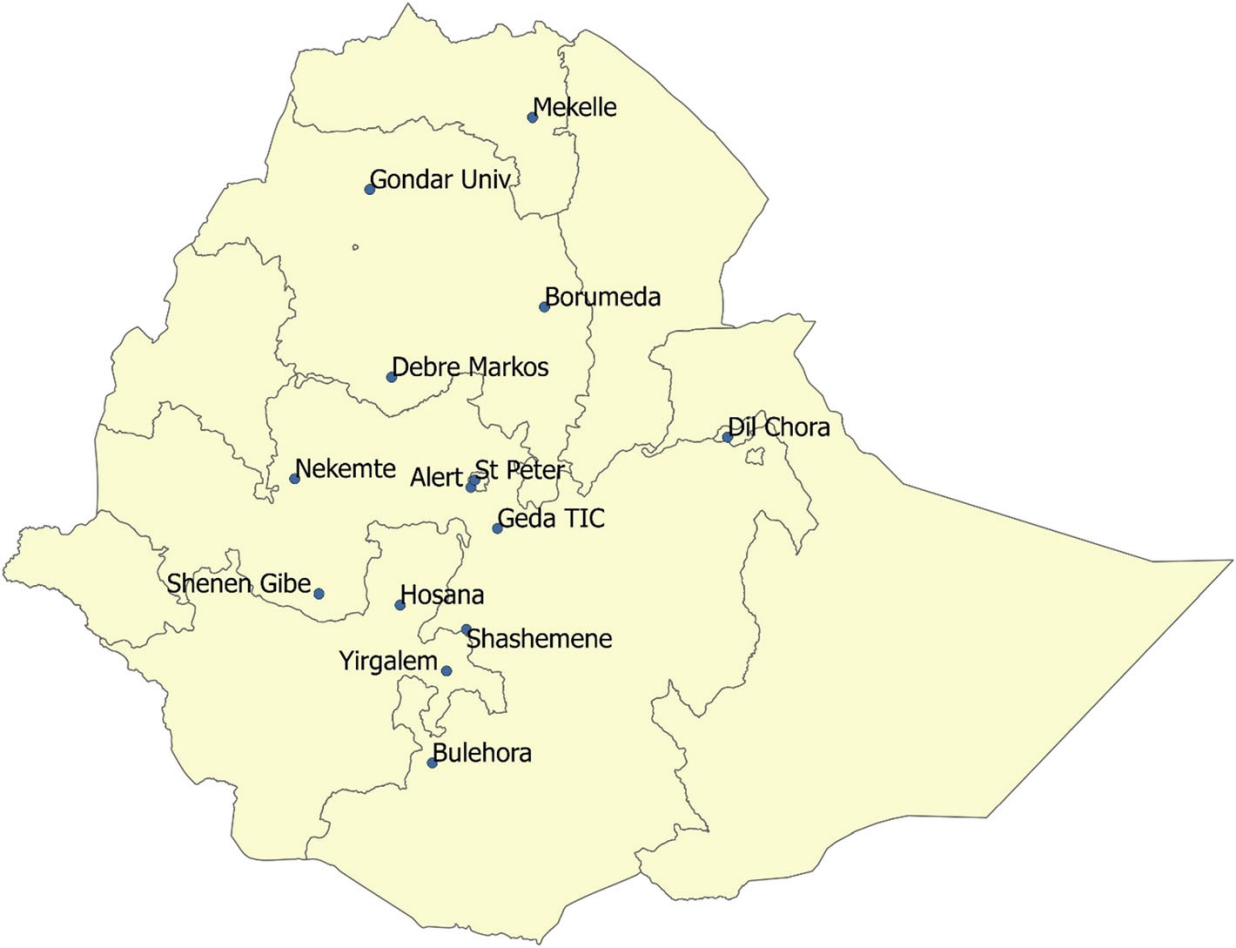
Map of Ethiopia with the location of MDRTB treatment initiation centres included in the study. Source: Federal Democratic Republic of Ethiopia Population Census Commission Bureau

The study was conducted using records of a random subset of the patients who started MDR-TB treatment between 2009 and 2014 (Fig. 1).

### Study design and population

A health facility-based retrospective cohort study design was used. The study population was all pulmonary MDR-TB patients who started treatment between January 1, 2009 and December 31, 2014 in all MDR-TB TICs in Ethiopia. Confirmed pulmonary MDR-TB patients based on culture and DST or Genexpert or Line Probe Assay and with positive base line culture were included in the study. Patients who transferred in were excluded from the sample.

### Sample size and sampling techniques

The sample size was calculated considering a 95% Confidence level (Zα/2) at 1.96; 31.6% unfavorable treatment outcome (p) [18]; 2.5% degree of precision (d); total study population (N) = 1559 and finite population correction [19]. Based on this, the calculated sample size was 751.

### Sampling technique

**Fig. 2:**
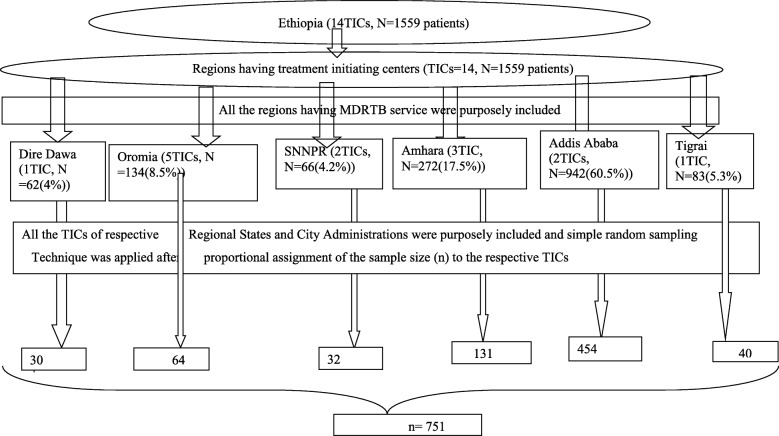
Schematic diagram of sampling technique for MDR/RR-TB patients treated in Ethiopia, 2009–2014. Legend: SNNPR- Southern Nations and Nationality Peoples’ Region; TICs-Treatment initiating centers; N-study population; n-sample size

All the 14 MDR-TB TICs found in Ethiopia from 2009 to till the end of 2014 were included in the study. These TICs were dispersed over the country with 1 found in Tigrai, 3 in Amhara, 5 in Oromia, 2 in Southern Nations and Nationality Peoples’ Regional States (SNNPR), 2 in Addis Ababa and 1 in Dire Dawa City Administrations [6]. The sample size was proportionally distributed over each TIC based on their patient load. These 14 TICs also hosted patients from the other five regions in the country that did not have TICs. A sampling frame for each TIC was prepared to select the 751 patients using the simple random sampling method from the MDR-TB register. The sampling procedure is pictured in Fig. 2.

### Measurements

The main outcome variable of this study was treatment outcome (categorized as favorable, unknown or unfavorable). The independent variables included were socio-demographic characteristics (age, sex and place of residence) and clinical conditions [type or form of TB (smear positive or smear negative TB), HIV/AIDS status, category of TB patients (new, return after lost to follow up, treatment failure, relapse and other), weight, presence of a TB treatment supporter, treatment regimen (new versus retreatment), having co-morbidities other than HIV/AIDS, BMI, bacilli load, degree of drug resistant and X-ray findings].

### Operational definitions /definition of terms

#### MDR-TB

A strain resistant to at least Rifampicin or both Rifampicin and Isoniazid.

#### Intensive phase treatment outcome

The outcome at which injectable agent was discontinued and the patient put on an oral continuation regimen.

#### Favorable treatment outcome

The outcome at which the patient was culture converted and alive at the end of the intensive phase.

#### Unknown treatment outcome

The outcome at which sputum culture not done or sputum culture sample sent but no feedback or no result or information to assign culture converted or not.

#### Unfavorable treatment outcome

Included lost to follow up, died, not evaluated, treatment terminated and culture not converted at the end of the intensive phase.

#### Sputum conversion

Defined as two consecutive negative smears or cultures from samples collected at least 30 days apart.

### Data collection

A pretested and structured record review checklist was used to collect the data from the MDR-TB register and treatment card. Where needed, the ART register was reviewed to complete missing information from the MDR-TB register for those HIV positive patients enrolled to chronic HIV/ART care. Data were collected by two teams each consisting of one supervisor and two data collectors. The data collectors were trained nurses and the supervisors were trained BSc public health graduates. To maintain data quality, the collected data were submitted daily to the supervisors for verification with feedback provided the following morning.

### Statistical analysis

Data were entered into and cleaned using Epidata version 3.02 and analyzed using Statistical Package for Social Sciences (SPSS) version 20.0. Exploratory data analysis was carried out to check the level of missing values and presence of influential outliers. Multi-co linearity, normality was also checked for continuous variables. The normality of the data was checked using a histogram. For continuous and normally distributed data mean and standard deviation were reported and otherwise median and inter quartile range were reported. For categorical variables, frequencies and percentage were reported. The median duration of the intensive phase treatment and the median time of sputum culture and smear conversion were computed.

The association between the independents and dependent variables was performed first with bivariate multinomial logistic regression analysis with relative risk ratio (RRR) at 95% confidence interval and favorable, unknown and unfavorable treatment outcomes were reported.

Finally, multivariable multinomial logistic regression analysis was done to identify independent factors associated with MDR-TB intensive phase treatment outcomes. For the purpose of selecting potential candidate variables, a multivariable model was constructed for variables having a *P* value < 0.25 in the bivariate analysis [20]. Statistical significance was considered with two sided *P*-values of 0.05 and 95% Confidence Intervals (CI).

## Results

### Socio-demographic characteristics

In this study, a total of 751 MDR-TB patient’s records were reviewed. The median age of the patients was 28 (IQR 23–38) years. Majority of the patients were urban dwellers (67.6%) and males (57.7%). Over the six year period, a total of 454 (60.5%) of patients were treated at TICs in Addis Ababa City Administration (AACA) of which 181 (40%) of the patients came from Regional States other than AACA. The majority of patients, 376 (50%), were treated at St. Peter Hospital TIC, the first TIC established in the country, followed by Gondar University Hospital TIC with 93 (12.4%) of the patients (Table 1).

**Table 1:** Socio-demographic characteristics of Multi-drug resistant tuberculosis (MDR-TB) patients treated in Ethiopia, between 2009 and 2014 (*N* = 751)

Variables	Frequency	Percent (%)
Regions at which the patients treated:		
Addis Ababa	454	60.5
Amhara	131	17.4
Oromia	64	8.5
Tigrai	40	5.3
SNNPR	32	4.3
Dire Dawa	30	4
Region from where the patients were come:		
Addis Ababa	273	36.4
Amhara	144	19.2
Oromia	140	18.7
SNNPR	70	9.4
Tigrai	59	7.9
Dire Dawa	25	3.4
Afar	10	1.3
Somali	10	1.3
Harari	3	0.4
Gambela	2	0.3
Benishangul Gumuz	2	0.3
Unknown	13	1.8
TIC at which the patients treated treatment		
St.Peter hospital	376	50
Gondar university hospital	93	12.4
ALERT hospital	78	10.4
Mekele hospital	40	5.4
Borumeda hospital	34	4.6
Dilchora hospital	30	4
Shashamane hospital	13	1.7
Nekemte hospital	19	2.5
Adama hospital	17	2.3
Bulehora hospital	2	0.3
Shenengibe hospital	13	1.7
Yergalem hospital	16	2
Hosana hospital	16	2
Debremarikos hospital	4	0.5
Sex		
Male	433	57.7
Female	318	42.3
Residence:		
Urban	506	67.4
Rural	245	32.6

### Clinical/programmatic characteristics

From the 751 records reviewed, 563 (75%) patients were treated under the hospitalized model of care. About two-in-five, 293 (39.0%) had a history of at least one co-morbidity with 164 (21.8%) being HIV co-infected. Only 455 (60.6%) had recorded information to calculate the BMI. Among them, 314 (69%) had a BMI below 18.5 kg/m^2^, the cut of point to define under nutrition, while the median BMI was 16.65 (IQR14.80–19.20) kg/m^2^.

From the total sample, 542 (72.2%) patients were sputum smear positive at month zero. From these, sputum grading or bacilli load data was available for 396 (73%) only. Almost all patients (97%) were previously treated for TB with nearly three quarters (73.9%) of them after treatment failure. All of the included patients were resistant to Rifampicin. With regards to potassium (K^+^) and X-ray findings, 45.1% had history of hypokalemia and 90.6% had abnormal X-ray findings (90.6%) (Table 2).

**Table 2:** Clinical and programmatic characteristics of the patients treated for multi-drug resistant tuberculosis in Ethiopia between 2009 and 2014 (*N* = 751)

Variables	Frequency	Percent (%)
Model of care		
Ambulatory	178	23.7
Hospitalized	573	76.3
TB treatment supporter		
Yes	399	53
Missing/unknown	43	6
No	309	41
Type of TB treatment supporter		
HCW	19	4.8
Family	359	90
Other	21	5.2
Co-morbidity (at least one)		
Yes	293	39
Unknown	65	8.7
No	393	52.3
HIV status		
Reactive	164	21.8
Non-reactive	587	78.2
Diabetes mellitus		
Yes	26	3.5(*6.7)
No	361	48(*93.3)
Unknown	364	48.5
Body mass index (kg/m^2^) at start of treatment		
< 18.5	313	41.8
Unknown	296	39.4
> =18.5	142	18.8
Treatment interruption during treatment		
Yes	28	3.7
No	669	89.1
Missing	54	7.2
Medication changed		
No	248	33
Unknown	432	57.5
Yes	71	9.5
Reason for medication changed		
Side effect	62	87.3
National algorithm change	3	4.2
Others	6	8.5
Pulmonary tuberculosis		
Smear positive	542	72
Smear negative	209	28
Bacilli load		
Scanty	15	2.8
+	119	22
++	155	28.6
+++	107	19.7
Unknown	146	26.9
Previous history of susceptible TB treatment		
Yes	730	97.2
No	21	2.8
Frequency of susceptible TB treatment		
One	158	21.6
Two	400	54.8
> =Three	172	23.6
Resistance to		
Four drugs (RHES)	167	22.2
Three drugs (RES/RHE)	57	7.6
Only two drugs (RH)	366	48.8
Only one drug (R)	161	21.4
Category of the patient at start of treatment		
Failure	555	73.9
Relapse	70	9.3
LTFU & Other	105	14
New	21	2.8
History of hypokalemia		
Yes	341	45.4
Missing/unknown	90	12
No	320	42.6
Treatment outcome of FL treatment		
Unsuccessful	645	88.4
Successful	85	11.6
Base line x-rays done		
Yes	470	62.5
No	148	19.7
Missing	133	17.8
X-ray result/finding		
Abnormal	426	90.6
Unknown	30	6.4
Normal	14	3
History of second line drug		
Yes	15	2
No	736	98

*Percent from those with available information on history of DM

*TB* = Tuberculosis=, *HCW*=Health Care Worker, *HIV*=Human Immune Deficiency Virus, *FL* = First line, *DM* = Diabetes Mellitus

### Treatment outcomes

**Fig. 3:**
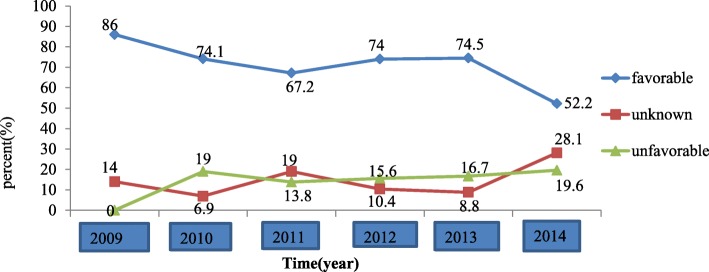
Trends of intensive phase treatment outcome among patients treated for MDR/RR-TB in Ethiopia, 2009–2014

With regards to outcome, 130 (17.3%) of the patients had an unfavorable, 126 (16.8%) had an unknown and 495 (65.9%) had a favorable treatment outcome. The trend of favorable treatment outcome declined from 2009 to 2011; but showed a slight increment from 2012 to 2013. The unfavorable treatment outcome increased during the year 2010 and became constant thereafter. The median time from diagnosis to treatment initiation of MDR-TB was 2.96 months (IQR = 0.73–7.24). The median duration of the intensive phase of MDR-TB treatment was 9.01 months (IQR 8.04–10.54) (Fig. 3).

### Sputum smear and culture conversion

**Fig. 4:**
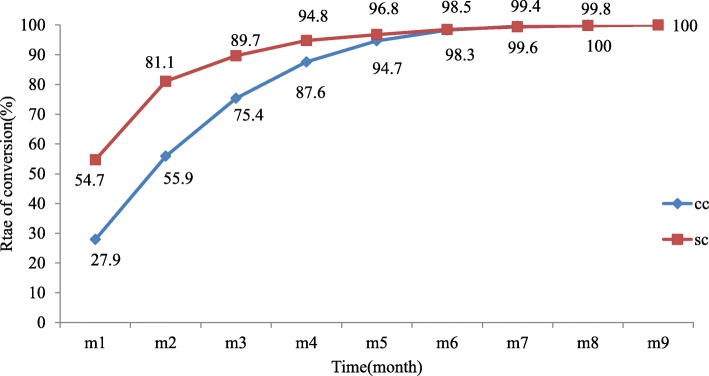
Rate of sputum smear and culture conversion among patients treated for MDR-TB in Ethiopia, 2009–2014. Legend: M-month; CC-culture conversion; SC-smear conversion

At month Zero, 542 (72.2%) patients were known sputum smear positive of whom complete data to determine rate of sputum smear conversion was available for 466 (86%). Of these 466 patients, 255 (54.7%) converted to sputum smear negative at month one and 378 (81%) converted to sputum smear negative at month two. From the 751 initial sputum cultures positive patients, complete data were available for 524 (69.8%) to determine rate of sputum culture conversion. Of these, 146 (27.9%) converted at month one and 293 (55.9%) converted at month two. The median duration of sputum smear conversion was 1 month (IQR =1–2) while for culture conversion this was 2 months (IQR = 1–3) (Fig. 4).

### Factors associated with intensive phase treatment outcome

In bivariate analysis, the co-variates with *p*-value less than or equal to 0.25 of level of significant for unknown treatment outcome were age, residence, model of care, TB treatment supporter, history of TB treatment interruption, history of medication changed, sputum smear positive, bacilli load, liver function test, number of abnormal X-rays findings, category of the patient, previous history of FLD and SLD TB treatment, degree of drug resistance, history of hypokalemia and year of treatment initiation.

Whereas age, comorbidity, BMI, treatment outcome of first line TB, category of the patient, history of treatment interruption, HIV status, type of TB treatment supporter, model of care, degree of drug resistance, history of hypokalemia, liver function test and year of treatment initiation were statistically significant at 0.25 level of significant for unfavorable treatment outcome.

In multivariable analysis, model of care, residence, TB treatment supporter and degree of drug resistance were statistically significant for unknown outcome at 0.05 level of significant. For unfavorable treatment outcome age and serum potassium level were statistically significant at 0.05 level of significant and included in the final model (Table 3).

**Table 3:** The independent predictors of unknown and unfavorable intensive phase treatment outcome among patients treated for MDR-TB in Ethiopia, 2009–2014

Variables	Unknown outcome	
	CRRR 95% CI	ARRR 95% CI
Model of care		
Ambulatory	2.107 (1.386, 3.203) **	3.158 (1.612, 6.185) **
Hospitalized	1	1
Residence		
Urban	0.538 (0.361,0.803) **	0.370 (0.199, 0.66) **
Rural	1	1
TB Treatment supporter		
Yes	0.964 (0.634, 1.465)	0.022 (0.002, 0.231) **
Unknown	3.383 (1.632, 7.012) **	0.332 (0.083, 1.319)
No	1	1
Resistances to:		
Four drugs (RHES)	0.293 (0.165,0.523) **	0.741 (0.267, 2.059)
Three drugs (RHE)	0.254 (0.106,0.609) **	0.185 (0.03, 0.9426) *
Two drugs (RH)	0.285 (0.178,0.457) **	0.350 (0.177, 0.693) **
One drug(R)	1	1
	unfavorable outcome	
	CRRR 95% CI	ARRR 95%CI
Age	1.039 (1.022, 1.056) **	1.047 (1.024, 1.072) **
History of hypokalemia		
Yes	0.567 (0.371, 0.867)**	0.512 (0.280, 0.939) *
Unknown	1.548 (0.857, 2.797**)**	1.454 (0.499, 4.242)
No	1	1

*CRRR* = Crude Relative Risk Ratio, *ARRR* = Adjusted Relative Risk Ratio, *p value< 0.05;

**P value< 0.01

## Discussion

Our study showed that about two third, 495 (65.9%) of patients had a favorable outcome at the end of intensive phase, whereas 130 (17.3%) had an unfavorable; and 126 (16.8%) had an unknown treatment outcome. The median duration of intensive phase treatment was 9.0 months. Being older and hypokalemic were associated with unfavorable intensive phase treatment outcome whereas, having been treated under the ambulatory model of care, being a rural dweller, not having a treatment supporter and lower degree of drug resistance were factors associated with unknown treatment outcome.

The favorable treatment outcome trend sharply declined from 86% in 2009 to 67.2% in 2011 and increased again slightly to 74% in 2013 but showed again a sharp decline to 52% in 2014. The trend is largely affected by those with an unknown outcome, especially in the year 2014. A possible explanation for the flactuation could be the shift to the ambulatory (decentralized) model of care since 2013/14 and lack of access to culture result due to limited mumber of culture facilitities in the peripheral part of the country.

The proportion of patients with an unfavorable treatment outcome in this study was 17.3%. This was similar to studies conducted in Nigeria (15%) [21] and Botswana (16, 15 and 17% among overall, HIV positive and HIV negative MDRTB patients respectively) [22]. Though our findings showed higher proportion of unfavorable treatment outcome than a study conducted in Tanzania (11%) [22], but lower than studies reported in South Africa (31.6%) [18], China (26.6%) [23] and India (38%) [24]. The observed differences might be due to expansion of the ambulatory model of care in Ethiopia, also the other studies did not consider unknown treatment outcome, a key driver of our trend and there were also other differences like sample size, study period and study setting. For instance, the studies in Tanzania and Nigeria [21, 25] were conducted over shorter periods of 3 years (2011–2012 and 2009–2011, respectively) while our study included 6 years data (2009–2014), twice as long. The Tanzanian study included only data on the hospitalized model of care. However, our study included both the hospitalized and ambulatory model of care.

The median duration of the intensive phase treatment in this study was 9 months (IQR = 8.0–10.5). This duration is longer than the 7 months (IQR = 6–8) reported from Tanzania [25]. This could be due to differences in the case definition of the duration of the intensive phase. For example, in Tanzania it was defined as 8 months **OR** 4 months after culture conversion. However, in Ethiopia, it is defined as at least 8 months **AND** 4 months after culture conversion, whichever is longer. Inadequate access to culture facilities at the peripheral part of the country could also contribute to the length of this period as often the duration of the intensive phase is decided upon clinically by a panel team and if culture results are not available timely this may result in longer durations of the intensive phase.

As age increases by one year (older age), the likelihood of experiencing unfavorable treatment outcome increased by 1.047(ARRR = 1.047:1.024, 1.072). Similar findings were seen in studies from Peru, Latvia, Estonia, Russia and the Philippines who reported that older age was associated with less likelihood of culture conversion [13].

Serum potassium level was a factor significantly associated with unfavorable treatment outcome; with having a history of hypokalemia decreasing the risk of unfavorable treatment outcome by 49% (*p-*value 0.031) compared with those who did not have a history of hypokalemia. This finding is counterintuitive and needs further exploration.

The proportion of patients with an unknown treatment outcome in this study was 16.8% and fluctuated over the study period (14–28.1%) showing an overall increase over time. Other studies did not include those with unknown outcome. If we take these out of the analysis the overall proportion with favorable outcome is 79.2% and those with unfavorable is 20.8%.

Those patients who had been treated under the ambulatory model of care were 3.2 times more likely (ARRR = 3.2:1.612, 6.185) to have an unknown treatment outcome. This might be due to the fact that this is relatively decentralized and there are a limited number of culture facilities in the peripheral part of the county. This may result in delayed feedback of culture result or not sending a sample for culture resulting in unknown outcome. This needs further exploration as it is important that as per guideline all patients have all required specimen taken and analyzed to allow for proper outcome monitoring and correct treatment.

Place of residence was also associated with unknown treatment outcome. For urban dwellers the likelihood of having unknown treatment outcome decreased by 63% (*p*-value< 0.01) compared to rural dwellers. This may be due to the distance patients reside from the facilities which may lead to non-adherence to scheduled laboratory monitoring. Or the urban TICs may have better recording practice.

Patients resistant to two or three drugs were less likely to have an unknown treatment outcome compared to those resistant to a single drug. This finding was unexpected and needs further exploration. It may be that those patients with multiple drug resistant were given closer follow up and patients diagnosed by Genexpert had information only about rifampicin resistant even though the patients might be resistant to other drugs besides rifampicin which might mask the true findings.

The high levels of unfavorable and unknown treatment outcomes have impacts on quality of life and transmission of MDR-TB in community. This might also prone an individual towards extensive drug resistance TB.

The study has strengths and limitations. Being a national representative data set and the large sample size are clear strengths. In addition, including unknown outcome as done in this study is important to fully understand the picture of early treatment outcome and does provide important insight for the TB prevention and control program. Limitations are that we did not consider multilevel modeling to understand the regional Variation in terms of different factors.

## Conclusion

The intensive phase favourable treatment outcome continues to decline in Ethiopia with an increase in unfavorable and unknown treatment outcomes most likely after the implementation of the ambulatory model of care which requires attention. Ambulatory model of care, rural dwellers, not having treatment supporter and limited number of drug resistance were associated with unknown treatment outcome. While age and serum potassium levels were associated with unfavourable treatment outcome. In order to minimize the unknown and unfavorable outcomes and have complete data for in-depth analysis, health care providers working in the different treatment initiating centers should adhere to the scheduled laboratory monitoring test especially culture, drug susceptibility test and serum potassium level and track the culture result feedback as early as possible and ensure all available data are duly recorded. Health care providers should also closely monitor older aged patients, perform regular death audits, trace those lost to follow up and ensure that treatment supporters are well trained/oriented and every patient is assigned one.

The Federal Ministry of Health and Regional Health Bureau should strengthen the sample referral system to increase access to culture and drug susceptibility testing for patients and ensure the feedback system is working optimally especially for the ambulatory model of care and rural residents. Further, a prospective study including primary data and multilevel modeling in order to explore additional contributing factors to the intensive phase treatment outcome at all levels of the treatment initiating centers could gain further necessary insights. Moreover, a study should be conducted on the final treatment outcome for the same study subjects to see whether similar outcomes were achieved or not.

## Data Availability

All the necessary data supporting our findings are contained within the manuscript. The datasets used and/or analyzed during the current study available from the corresponding author on reasonable request.

## Abbreviations

AFB: Acid Fast Bacilli
AIDS: Acquired Immune Deficiency Syndrome
ARRR: Adjusted Relative Risk Ratio
ART: Antiretroviral Therapy
BMI: Body Mass Index
CPT: Cotrimoxazole Preventive Therapy
CSA: Central Statistical Authority
DR-TB: Drug Resistant Tuberculosis
FDRE-PCC: Federal Democratic Republic of Ethiopia- Population Census Commission
FMOH-E: Federal Ministry of Health of Ethiopia
HBCs: High Burden Countries
HC: Health Center
HIV: Human Immune Deficiency Virus
HR: Isoniazid and rifampicin
HRES: Isoniazid, Rifampicin, Ethambutol and Streptomycin
HRS: Isoniazid, Rifampicin and Streptomycin
IQR: Inter Quartile Range
MDR(X)-TB: Multidrug resistant-TB or Extremely Drug Resistant-TB
MDR-TB: Multidrug Resistant Tuberculosis
MTB: *Mycobacterium tuberculosis*
NA: Not applicable
PTB: Pulmonary Tuberculosis
SLD: Second Line Drug
SNNPR: Southern Nations and Nationality Peoples’ Region
SPSS: Statistical Package for Social Sciences
TB: Tuberculosis
TBL: Tuberculosis and Leprosy
TFC: Treatments Follow up Center
TIC: Treatment Initiating Center
TRAC: Tuberculosis Research Advisory Committee
TSR: Treatment Success Rate
UNAIDS: United Nations Joint Program on HIV/AIDS
UNICEF: United Nations International Children’s Fund
WHO: World Health Organization
